# Comparative analysis of prokaryotic and eukaryotic transcription factors using machine-learning techniques

**DOI:** 10.6026/97320630014315

**Published:** 2018-06-30

**Authors:** Chowdhury Nilkanta, Angshuman Bagchi

**Affiliations:** 1 Department of Biochemistry and Biophysics, University of Kalyani, Kalyani, Nadia 741235, India

**Keywords:** Prokaryotic and Eukaryotic Organisms, DNA binding proteins, Transcription factors, Distribution of amino acid residues

## Abstract

The DNA-protein interactions play vital roles in the central dogma of molecular biology. Proper interactions between DNA and protein would lead to the onset of various biological phenomena like transcription, translation, and replication. However, the mechanisms of these well-known processes vary between prokaryotic and eukaryotic organisms. The exact molecular mechanisms of these processes are unknown. Therefore, it is of interest to report the comparative estimate of the different properties of the DNA binding proteins from prokaryotic and eukaryotic organisms. We analyzed the different sequence-based features such as the frequency of amino acids and amino acid groups in the proteins of prokaryotes and eukaryotes by statistical measures. The general pattern of differences between the various DNA binding proteins for the development of a prediction system to discriminate between these proteins between prokaryotes and eukaryotes is documented.

## Background

DNA protein interactions as in DNA transcription are at the heart of the central dogma of molecular biology. The transcription is the process of transfer of genetic information from DNA molecules. The process is regulated by a set of proteins. These proteins are referred to as the transcription factors (TFs) [1]. The mechanism of the process is a very complex one and is mainly mediated by a complex interplay between the TFs with DNA. However, the mechanism of DNA transcription is different in prokaryotic and eukaryotic organisms [2, 3].

However, the molecular details of the transcription processes in the pro- and eukaryotic organisms are still at its infancy. In this work, we tried to analyze the different aspects of the transcription factors from pro- and eukaryotic organisms. For the comparison purposes, we used the amino acid sequences of the DNA binding proteins (DBPs) and transcription factors (TFs) from UniProt [4].

We compared the TFs using their sequence information only as sequence is more abundant than structure [5]. The main motivation of carrying out the work is to discriminate between the different classes of microorganisms. We, for the first time, put forward some plausible discriminatory features between the TFs from the different branches of organisms. Interestingly, the TFs from the pro- and eukaryotic organisms can be distinctly identified using the amino acid frequency analyzes in the TFs. We also analyzed the statistical efficacies of the features used in the study to discriminate between the different classes of microorganisms using machine-learning techniques. The ideas regarding these features may further be utilized to come up with a prediction system to discriminate between the different branches of organisms.

## Methodology

### Data collection

**Figure 1:**
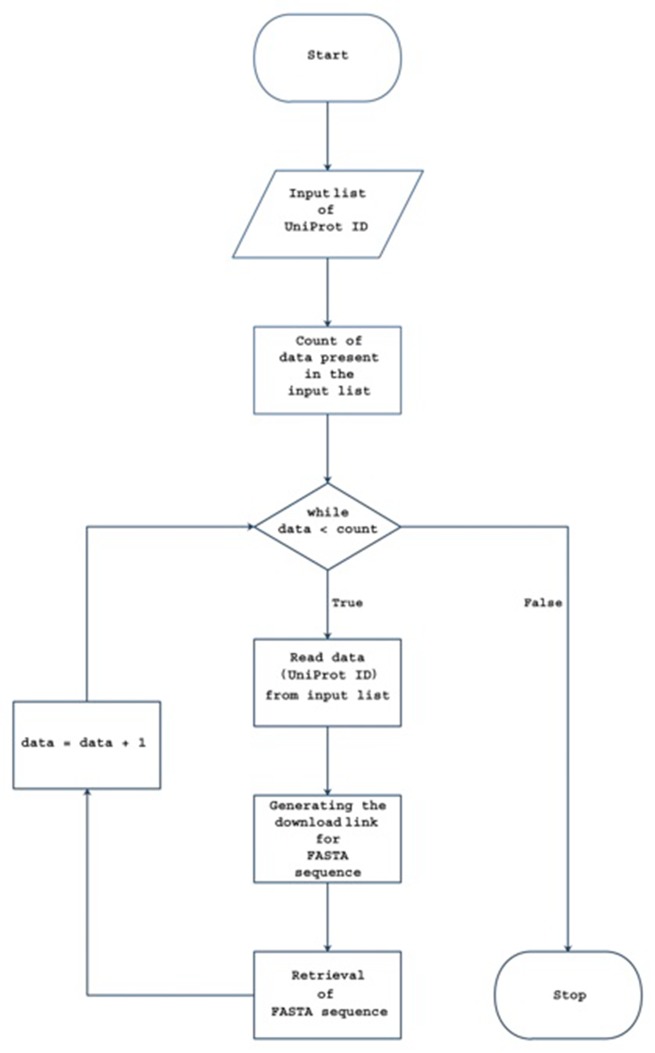
Flowchart diagram of the in-house python tool.

We downloaded the sequences of DNA binding proteins (DBPs) from UniProt [4]. We collected the amino acid sequences of the DNA binding proteins from 1012 prokaryotic organisms and 1425 eukaryotes. We divided our dataset into two groups, the largest group containing the whole DBP data, and a small subgroup containing the transcription factor (TF) sequences, which were also present in the DNA binding protein dataset. The data collection process was carried out using an in-house tool written in Python (Figure 1).

### Redundancy check to the dataset

The raw dataset may be biased because of having multiple copies of a single sequence. We, therefore, performed a redundancy check, by means of distance matrix calculation. The distance matrix was generated by Hamming distance algorithm [6, 7]. After this redundancy check, we were able to eliminate the redundancy in the dataset and prepared a clean dataset. The clean dataset contained 270 DBP sequences from prokaryotes and 347 DBP sequences from eukaryotes; among them, there were 92 sequences of TF from prokaryotes and 182 sequences of TF from eukaryotes. So the DBP dataset contained 270 prokaryotic and 347 eukaryotic sequences. As the eukaryotic DBP sequences were present in higher number than the prokaryotic DBP sequences, we had split the eukaryotic DBP sequences into two sets. Eukaryotic DBP set 1 contained sequences starting from 1 to 270 and eukaryotic DBP and set 2 contained sequences starting from 78 to 347 so that there were equal numbers of amino acid sequences in the datasets. For the same reason, the eukaryotic TF dataset was split into two sets. TF set 1 contained sequences starting from 1 to 92 and TF set 2 contained sequences starting from 91 to 182. Thus all the datasets were balanced. The distribution of the dataset is shown in Table 1. The list of UniProt IDs used in these datasets was present in Table 1 (see supplementary material).

### Frequency Calculation

After the preparation of these clean datasets, we performed amino acids and amino acids group frequency calculations. We categorized the amino acid groups into Hydrophobic (HB), Hydrophilic (HI), Charged (CR), Basic (BS) and Acidic (AC) [8]. This frequency calculation was done to normalize the dataset. The entire frequency calculation was done using an in-house python script. We had calculated the frequency of amino acids and amino acid groups separately for the two datasets DBP and TF, and separately for eukaryotic set1 and eukaryotic set 2.

### Machine learning using WEKA

We used the overall amino acid frequencies and amino acids group frequencies of the prokaryotic and eukaryotic organisms as features to distinguish between prokaryotic and eukaryotic organisms using the tool WEKA [9]. WEKA is a tool, containing a collection of machine learning algorithms, is commonly used in data mining problems in bioinformatics. We have used the Support vector machine (SVM) algorithm and the SMO classifier [10] with 10 fold cross-validation. The 10 fold cross validation is a kind of default test option of WEKA. It randomly splits the dataset into training and testing datasets and runs the test. It does this operation 10 times with random splitting of the input data into training and testing datasets. We prepared the input dataset for WEKA using data distribution as described in Table 1.

## Results

### Amino acids and amino acid group frequency

**Figure 2:**
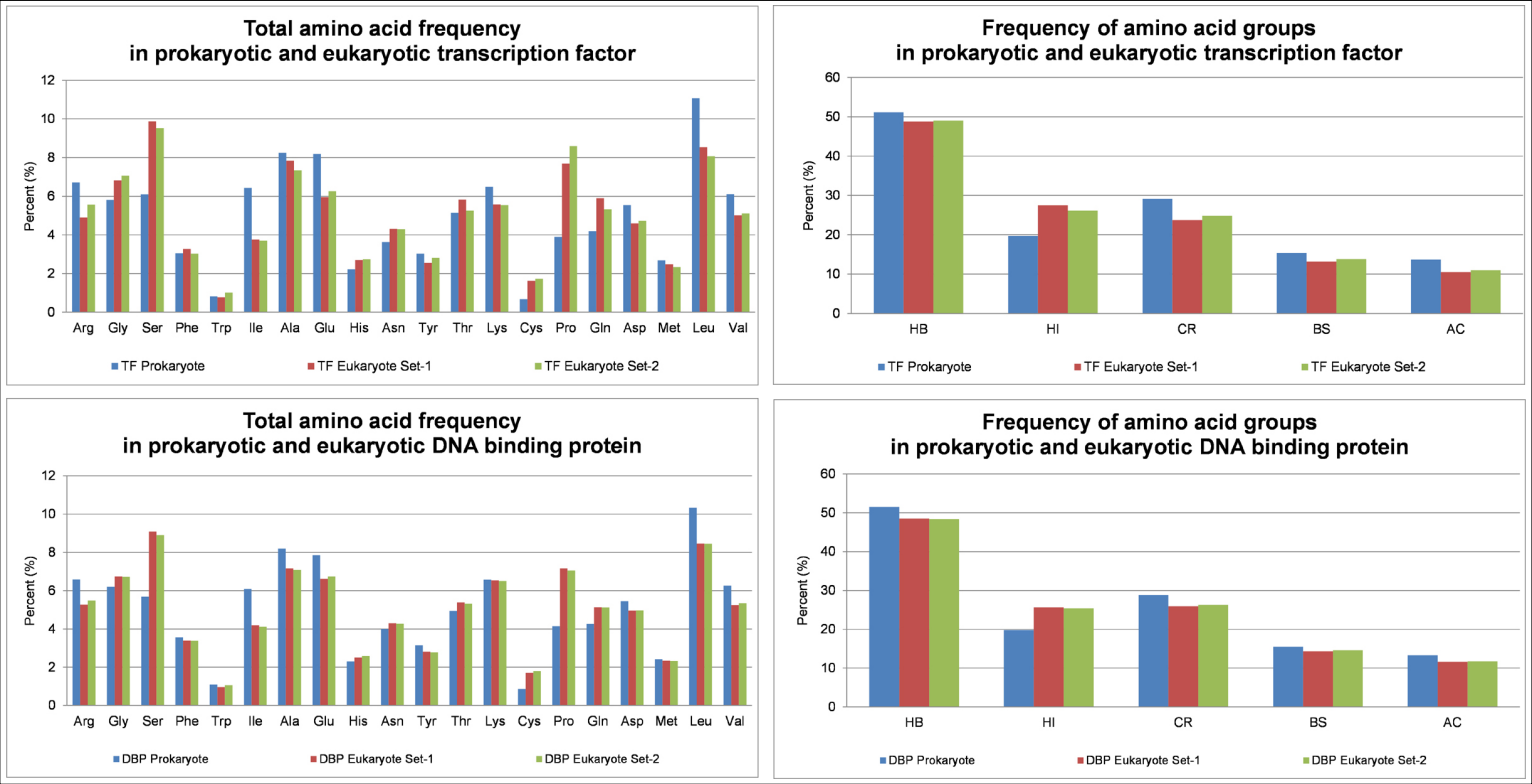
The bar-graph representation of amino acids and amino acid group frequency in prokaryotes and eukaryotes (Blue: Prokaryote; Red: Eukaryote Set-1; Green: Eukaryote Set-2).

**Figure 3:**
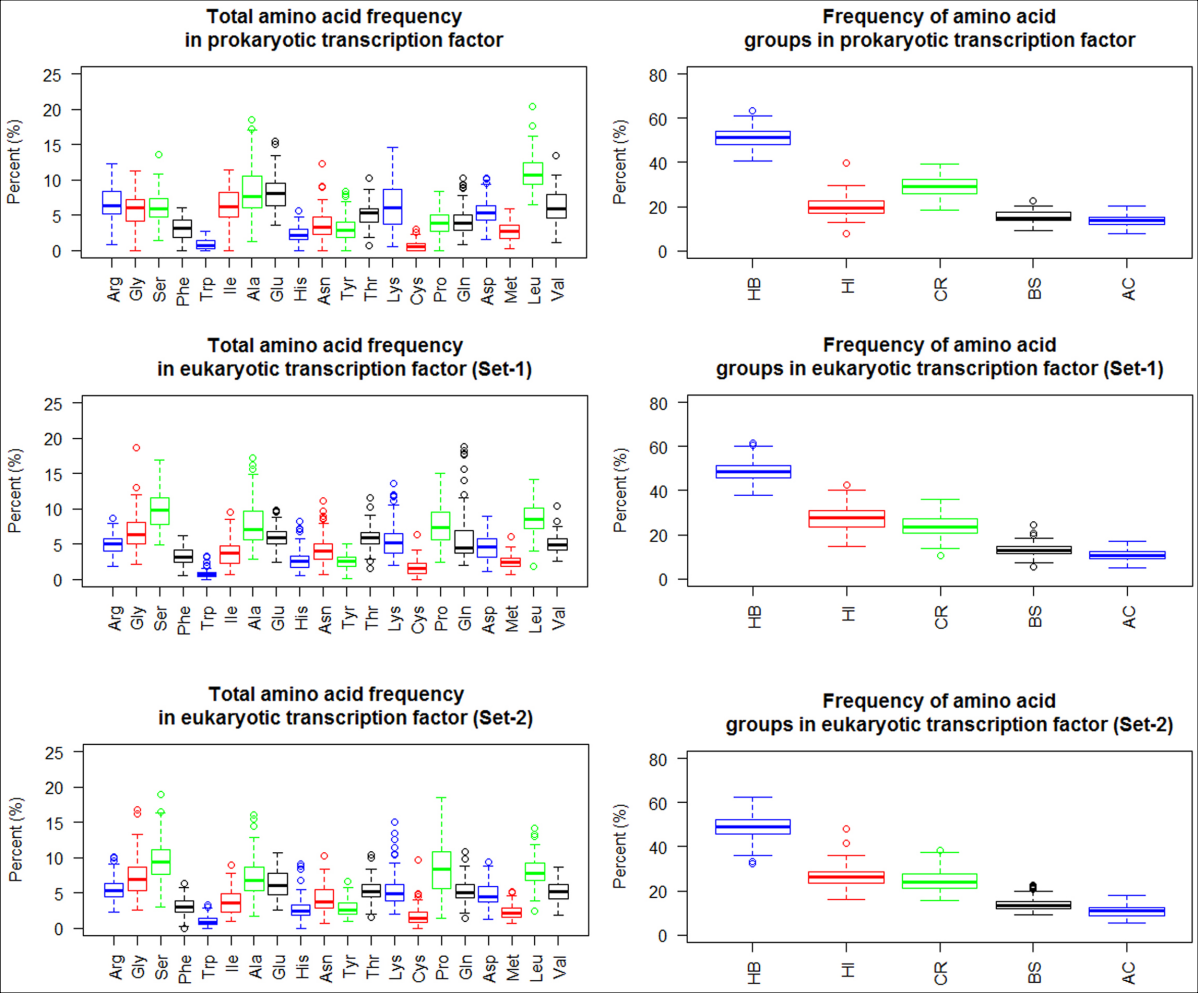
Amino acids and amino acid group frequency from TF dataset.

**Figure 4:**
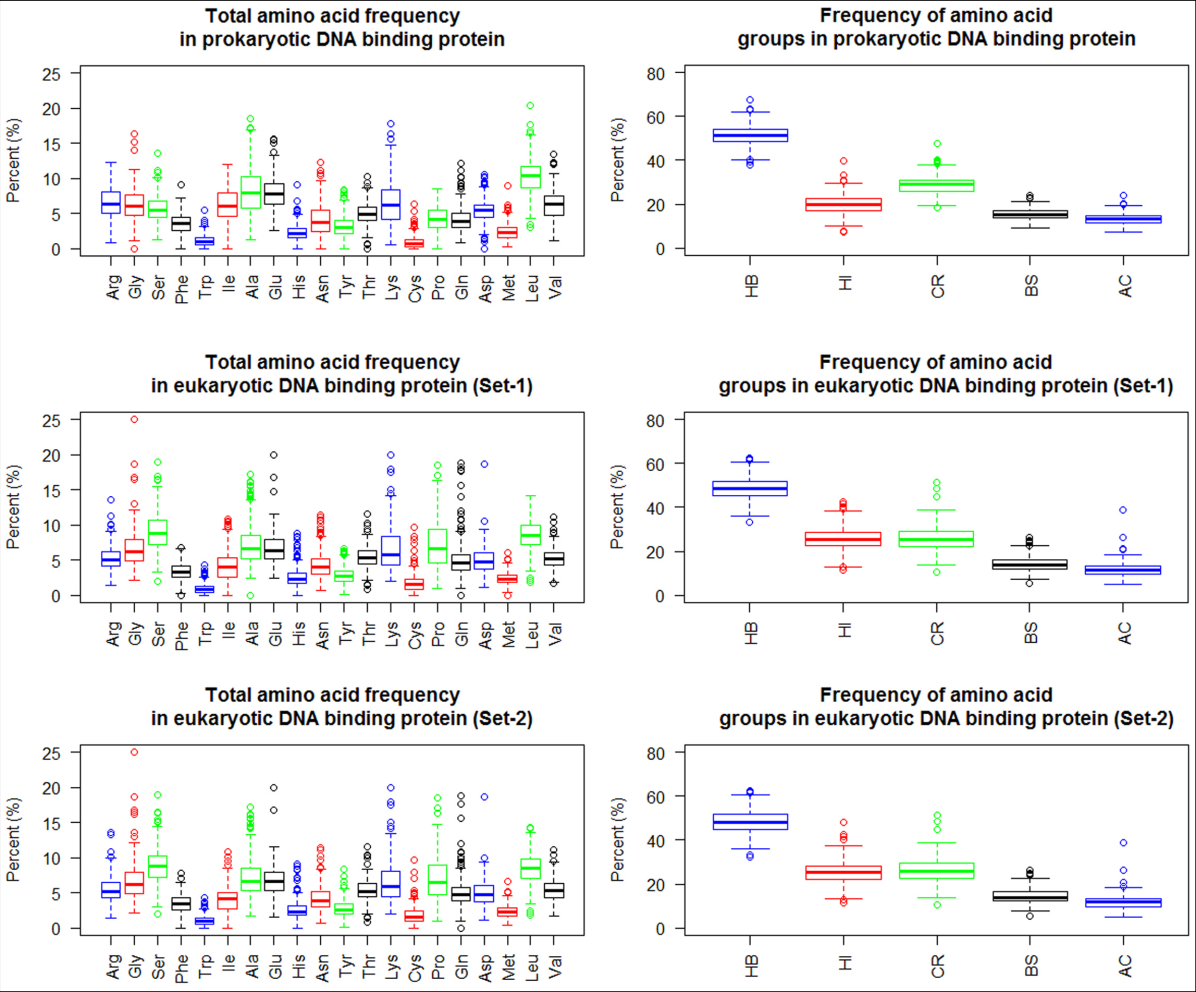
Amino acids and amino acid group frequency from DBP dataset.

A distinguishable difference was found in the frequency patterns between eukaryotic and prokaryotic amino acid sequences in the DNA binding proteins. This distinguishable difference pattern in amino acid and amino acid group frequency can be used to discriminate them. The bar graph (Figure 2) and boxplot (Figure 3 and Figure 4) were used to decipher the patterns of the differences.

### Machine learning results

We found that amino acids and amino acid group frequency can be used as features to train a SMO classifier in WEKA to distinguish prokaryotic and eukaryotic DNA binding proteins on the basis of their amino acid and amino acid group frequency as given in Table 2.

## Discussion

Data show that the sequence-based features of the DBPs and TFs could very well be used to distinguish between these classes of organisms. In all our analyses, we obtained an overall accuracy greater than 85% and an AUC value of 0.9. However, we had to use a comparatively small dataset due to paucity of data in the databases. None-the-less, this is the up to date data available till the date mentioned in the manuscript. Available predictors combine both the sequence and structural information for the discrimination purposes. Our predictor uses only sequence information and therefore may be considered a more general one as sequence information is more abundant than structural information. For extraction of the features, we used an in-house script written in python.

## Supplementary material

Data 1

## Figures and Tables

**Table 1:** The distribution of the dataset.

DNA Binding Protein (DBP) dataset		Transcription Factor (TF) Dataset	
Prokaryote 1 - 270	Eukaryote Set-1 1 - 270	Prokaryote 1-92	Eukaryote Set-1 1-92
	Eukaryote Set-2 78 - 347		Eukaryote Set-2 91 - 182

**Table 2:** Results obtained from WEKA analysis.

(Transcription Factor Set-1)									
Total Number of Instances					184				
Correctly Classified Instances					94.02%				
Incorrectly Classified Instances					5.98%				
=== Detailed Accuracy By Class ===									
TP Rate	FP Rate	Precision	Recall	F-Measure	MCC	ROC Area	PRC Area	Class
0.924	0.043	0.955	0.924	0.939	0.881	0.94	0.92	Prokaryot
0.957	0.076	0.926	0.957	0.941	0.881	0.94	0.908	Eukaryot
Weighted Avg.	0.94	0.06	0.941	0.94	0.94	0.881	0.94	0.914	

(Transcription Factor Set-2)									
Total Number of Instances					184				
Correctly Classified Instances					93.48%				
Incorrectly Classified Instances					6.52%				
=== Detailed Accuracy By Class ===									
TP Rate	FP Rate	Precision	Recall	F-Measure	MCC	ROC Area	PRC Area	Class
0.924	0.054	0.944	0.924	0.934	0.87	0.935	0.911	Prokaryot
0.946	0.076	0.926	0.946	0.935	0.87	0.935	0.902	Eukaryot
Weighted Avg.	0.935	0.065	0.935	0.935	0.935	0.87	0.935	0.907	

(DNA Binding Protein Set-1)									
Total Number of Instances					540				
Correctly Classified Instances					88.33%				
Incorrectly Classified Instances					11.67%				
=== Detailed Accuracy By Class ===									
TP Rate	FP Rate	Precision	Recall	F-Measure	MCC	ROC Area	PRC Area	Class
0.863	0.096	0.9	0.863	0.881	0.767	0.883	0.845	Prokaryot
0.904	0.137	0.868	0.904	0.886	0.767	0.883	0.833	Eukaryot
Weighted Avg.	0.883	0.117	0.884	0.883	0.883	0.767	0.883	0.839	

(DNA Binding Protein Set-2)									
Total Number of Instances					540				
Correctly Classified Instances					90%				
Incorrectly Classified Instances					10%				
=== Detailed Accuracy By Class ===									
TP Rate	FP Rate	Precision	Recall	F-Measure	MCC	ROC Area	PRC Area	Class
0.904	0.104	0.897	0.904	0.9	0.8	0.9	0.859	Prokaryot
0.896	0.096	0.903	0.896	0.9	0.8	0.9	0.861	Eukaryot
Weighted Avg.	0.9	0.1	0.9	0.9	0.9	0.8	0.9	0.86
