# Supplementary material for: Comparative analysis of prokaryotic and eukaryotic transcription factors using machine-learning techniques
Source: Bioinformation. 2018 Jun 30;14(6):315–26. doi: 10.6026/97320630014315 (PMC6137564; doi:10.6026/97320630014315)
Supplement: Data 1 [file 97320630014315S1.pdf]

BIOINFORMATION
Discovery at the interface of physical and biological sciences

Open access

Supplementary Data:

Table S1: List of UniProt id of the FASTA files used as dataset

Prokaryotic TF	Eukaryotic TF	Prokaryotic DBP	Eukaryotic DBP
A0A0H2VJZ8	A0AVK6	A0A072Z681	A0AVK6
A0QZ11	A2D9X4	A0A0H2VJZ8	A0JP82
A0R6I8	G0SB31	A0A0H2XIU6	A2D9X4
A6T8N1	G4NEJ8	A0QZ11	A5J036
B2SU53	L7I1M8	A0R6I8	A6ZL36
B8FW11	O00327	A3DJ38	B4F6I0
C3W947	O00482	A3FMN7	C0JWR6
D5KM69	O15350	A5TY69	C7SWF3
G3XCY4	O15409	A6T8N1	D2W6T1
O34777	O43435	B2MU09	D9IWL3
O34817	O43524	B2SU53	D9J034
O66551	O54790	B8FW11	E0YCK3
O66858	O94916	C1D7P6	F7WD42
O68014	O95238	C3W947	G0SB31
O69245	P01100	D4EMQ0	G4NEJ8
P03023	P01106	D5KM69	L7I1M8
P03052	P02340	D5MNX7	M1GSK9
P06533	P02833	D9N168	O00327
P06534	P02836	E1C9K5	O00482
P07674	P03001	G3XCY4	O13988
P0A0I7	P03069	O25100	O14770
P0A0N4	P03372	O25386	O14862
P0A247	P04150	O25758	O15350
P0A4T9	P04386	O25841	O15409
P0A6X7	P04637	O34777	O15527
P0A881	P05412	O34817	O43435
P0A8U6	P05554	O52512	O43524
P0A8V6	P05725	O66551	O54790
P0ACI0	P06536	O66659	O74859
P0ACJ8	P06601	O66858	O75362
P0ACP7	P06602	O68014	O75531
P0ACS2	P07270	O68557	O80358
P0ACT4	P07272	O68847	O82175
P0AF28	P08046	O69245	O94468
P0AFJ5	P08151	O83028	O94916
P0AG30	P08638	O87365	O95238
P0AGK8	P09077	O87963	O95243
P0C1U6	P09631	P00582	O95551
P0DJL7	P09956	P00642	P00639
P10026	P0CS82	P00648	P00734
P17893	P0CY08	P02958	P01100
P21866	P0CY10	P03004	P01106
P22262	P10037	P03013	P01127
P23873	P10085	P03018	P01837
P23874	P10276	P03023	P02263
P25144	P11473	P03052	P02340
P27709	P11831	P03067	P02833
P33905	P11938	P03856	P02836
P39075	P13297	P04390	P03001
P40676	P13393	P04395	P03069

BIOINFORMATION
Discovery at the interface of physical and biological sciences

Open access

P44558	P14859	P04995	P03372
P46828	P14921	P05050	P03870
P68261	P15036	P05102	P03880
P71039	P15207	P05327	P03882
P96711	P15806	P05523	P04150
P9WGZ1	P16236	P06134	P04275
P9WJB7	P17676	P06533	P04386
P9WME9	P17679	P06534	P04637
P9WMF8	P17789	P06612	P05231
P9WMH1	P18113	P07013	P05412
P9WMH3	P19419	P07674	P05554
P9WPY9	P19544	P08394	P05725
Q0P6M2	P19793	P09184	P06401
Q1D4I5	P19838	P09546	P06536
Q2ACK9	P20153	P09883	P06601
Q2FZ56	P20226	P09980	P06602
Q32WH4	P20263	P0A0I7	P06766
Q3ZD72	P20393	P0A0N4	P06786
Q45782	P20823	P0A247	P07199
Q46731	P21952	P0A459	P07270
Q46864	P22121	P0A4T9	P07272
Q57468	P22415	P0A6C1	P07276
Q5F882	P22670	P0A6R3	P08046
Q5Y812	P22829	P0A6Z6	P08151
Q746J7	P23511	P0A7C2	P08638
Q7AKF2	P23760	P0A7G6	P09077
Q7X0D9	P23772	P0A809	P09631
Q83TD2	P24781	P0A881	P09651
Q8AAV8	P25490	P0A8J2	P09838
Q8E565	P25502	P0A8U6	P09874
Q8GGH0	P25799	P0A8V6	P09884
Q8NMG3	P27577	P0A988	P09956
Q8YAF1	P28147	P0A9H1	P0CS82
Q933Z0	P28324	P0ABS5	P0CY08
Q9CHR1	P28347	P0AC51	P0CY10
Q9EZJ8	P29617	P0ACI0	P10037
Q9HUS3	P31266	P0ACJ8	P10085
Q9I1S1	P34707	P0ACP7	P10276
Q9KQU8	P35680	P0ACS2	P11308
Q9KWU8	P35869	P0ACT4	P11387
Q9S166	P36956	P0ADI2	P11473
Q9Z9H6	P38144	P0AEE8	P11831
	P38830	P0AEK0	P11938
	P38867	P0AF28	P12689
	P41235	P0AFJ5	P12956
	P42226	P0AFY8	P13051
	P42227	P0AG30	P13297
	P42582	P0AG74	P13393
	P43680	P0AGE0	P13864
	P46531	P0AGK8	P14585
	P47902	P0C1U6	P14653
	P48436	P0CI76	P14736
	P49711	P0DJL7	P14859
	P51608	P0DJO8	P14921
	P52952	P11405	P15036
	P53539	P13920	P15207
	P53762	P13925	P15424

P53999	P14294	P15436
P54841	P14385	P15806
P55318	P14565	P15919
P56178	P14633	P16236
P61244	P14870	P16455
P70118	P15005	P17255
P70340	P15042	P17542
P70348	P16525	P17676
P70512	P17743	P17679
P83949	P17888	P17789
P84022	P17893	P18113
P87249	P19821	P18858
P97360	P20384	P19419
P97471	P20589	P19544
P98177	P21189	P19793
Q00059	P21338	P19838
Q00403	P21866	P20153
Q00422	P22262	P20226
Q00613	P23478	P20263
Q00653	P23657	P20393
Q00958	P23873	P20823
Q01147	P23874	P21951
Q01167	P23909	P21952
Q01543	P23940	P22121
Q01663	P25144	P22415
Q01826	P27709	P22670
Q02078	P28630	P22829
Q02080	P30014	P23511
Q02548	P31032	P23760
Q03347	P33788	P23772
Q04206	P33905	P23906
Q04207	P37954	P24781
Q04863	P39075	P25490
Q05195	P40676	P25502
Q06330	P41016	P25799
Q06831	P42371	P26358
Q08050	P43642	P26367
Q08957	P43870	P26368
Q12778	P44558	P27577
Q13148	P44688	P27694
Q13469	P46828	P27695
Q14653	P50187	P28147
Q14863	P50465	P28324
Q14919	P52026	P28347
Q15561	P56255	P28519
Q16254	P56981	P29372
Q16666	P62558	P29549
Q17034	P68261	P29617
Q3UPW2	P70985	P31266
Q58HP3	P71039	P31483
Q5AP80	P72525	P31941
Q60793	P76116	P32657
Q61473	P83847	P32761
Q64249	P84131	P34257
Q6MZP7	P96711	P34707
Q6NT76	P96856	P35680
Q8C6P8	P9WGZ1	P35869

Q8GZB6	P9WII3	P36956
Q8IKH2	P9WJB7	P38144
Q8L7G0	P9WME9	P38830
Q8MXE7	P9WMF8	P38867
Q8NHW3	P9WMH1	P39748
Q94702	P9WMH3	P41235
Q94IF5	P9WNV3	P42224
Q95VR4	P9WPY9	P42226
Q969G2	Q031W6	P42227
Q99551	Q06B24	P42582
Q99626	Q0P6M2	P43246
Q9C932	Q1D4I5	P43680
Q9H3D4	Q2ACK9	P46531
Q9NQV7	Q2FZ56	P47902
Q9NUX5	Q2I6W2	P48436
Q9UHX1	Q32WH4	P49711
Q9UMN6	Q3ZD72	P49916
Q9Y5R6	Q45458	P50534
	Q45488	P50549
	Q45782	P51608
	Q46731	P52952
	Q46864	P53539
	Q46896	P53762
	Q46944	P53999
	Q47112	P54098
	Q47152	P54132
	Q47155	P54274
	Q47673	P54841
	Q47PJ0	P55265
	Q4UNB2	P55318
	Q53632	P56178
	Q56215	P60896
	Q57253	P61244
	Q57267	P61823
	Q57468	P61978
	Q5F882	P62805
	Q5F9M9	P63159
	Q5I6E6	P70118
	Q5KWC1	P70340
	Q5L0J3	P70348
	Q5SJ64	P70512
	Q5SJ65	P83949
	Q5SJC4	P84022
	Q5Y812	P87249
	Q72I39	P97360
	Q746J7	P97471
	Q746M7	P98177
	Q7AKF2	Q00059
	Q7CWV1	Q00403
	Q7DD47	Q00422
	Q7MHK3	Q00613
	Q7X0D9	Q00653
	Q816E8	Q00958
	Q83TD2	Q01147
	Q84AF2	Q01167
	Q8AAV8	Q01543
	Q8DPM2	Q01663

BIOINFORMATION
Discovery at the interface of physical and biological sciences

Open access

Q8E565	Q01826
Q8EFJ3	Q02078
Q8EIX3	Q02080
Q8EVR5	Q02486
Q8GGH0	Q02548
Q8KNP2	Q02880
Q8NMG3	Q03164
Q8R5T9	Q03347
Q8RNV5	Q04049
Q8RNV8	Q04206
Q8RT53	Q04207
Q8YAF1	Q04863
Q8Z2A5	Q05195
Q8ZG78	Q05783
Q928V6	Q06330
Q933Z0	Q06453
Q93PU6	Q06831
Q97FM4	Q07230
Q99U17	Q08050
Q9AC34	Q08874
Q9AFI5	Q08957
Q9AMH9	Q12778
Q9CHR1	Q13469
Q9EZJ8	Q13569
Q9F6L0	Q14191
Q9HUS3	Q14653
Q9I0M3	Q14863
Q9I1S1	Q14919
Q9I2N0	Q15109
Q9KEI9	Q15365
Q9KJ88	Q15366
Q9KQU8	Q15554
Q9KVD2	Q15561
Q9KWU8	Q16254
Q9KXR9	Q16531
Q9RPJ3	Q16666
Q9RT63	Q17034
Q9RWH8	Q25442
Q9RY80	Q3UPW2
Q9S166	Q4PRK9
Q9WY48	Q4VWW5
Q9WYV0	Q58HP3
Q9X2H9	Q5AP80
Q9X4C9	Q5EAW4
Q9XDH5	Q5NE14
Q9Z3B4	Q5XJA0
Q9Z9H6	Q60793
Q9ZL26	Q61473
V6F4Q0	Q64249
	Q68E01
	Q6CPM4
	Q6MZP7
	Q6N021
	Q6NS38
	Q6NT76
	Q6ZQJ5
	Q71DI3

Q7JQ07
Q7M3K2
Q7T2M9
Q7TS98
Q7Z2E3
Q7Z5Q5
Q84KJ5
Q84ZU4
Q86T24
Q8C6L5
Q8C6P8
Q8GZB6
Q8IKH2
Q8L7G0
Q8MXE7
Q8N5Y2
Q8NHW3
Q8SXK5
Q8SYK5
Q8VDF2
Q91VJ1
Q91XB0
Q921F2
Q92383
Q94702
Q94IF5
Q95VR4
Q969G2
Q96LI5
Q96LW4
Q96PU4
Q96T88
Q99551
Q99626
Q9C932
Q9DFY5
Q9GPZ9
Q9H171
Q9H3D4
Q9H9S0
Q9JIW4
Q9JJX7
Q9JLV6
Q9NP87
Q9NQV7
Q9NUW8
Q9NUX5
Q9P016
Q9P0U4
Q9QY24
Q9R002
Q9R1E6
Q9UBT6
Q9UBZ9
Q9UGP5
Q9UH17
Q9UHX1

BIOINFORMATION
Discovery at the interface of physical and biological sciences

Open access

	Q9UMN6
	Q9UNA4
	Q9UQ84
	Q9UTN9
	Q9VD99
	Q9VR17
	Q9Y253
	Q9Y261
	Q9Y2M0
	Q9Y5R6
	Q9YGN6
	Q9Z2D7
